# CRISPR/Cas9‐mediated editing of uORFs in the tryptophan decarboxylase gene *SlTDC1* enhances serotonin biosynthesis in tomato

**DOI:** 10.1111/pbi.70106

**Published:** 2025-05-31

**Authors:** Shiyang Zhang, Lei Zhu, Qingfeng Niu, Yansha Li, Xiaomu Niu, Jian‐Kang Zhu, Zhaobo Lang

**Affiliations:** ^1^ Institute of Advanced Biotechnology and School of Medicine Southern University of Science and Technology Shenzhen China; ^2^ Shandong Shunfeng Biotechnology Co. Ltd. Jinan China; ^3^ Research Center for Biological Breeding, Advanced Academy Anhui Agricultural University Hefei China

**Keywords:** serotonin, uORF, gene‐editing, Tomato, TDC1

Tomatoes (*Solanum lycopersicum*) are a unique fruit vegetable table combination, widely consumed for their rich bioactive compounds, including phenolic antioxidants and serotonin (5‐hydroxytryptamine, 5‐HT) (George *et al*., 2004). Serotonin has potential therapeutic applications for metabolic disorders such as hyperlipidemia, diabetes, and obesity (Jia *et al*., 2024; Oh *et al*., 2015). Tryptophan decarboxylase (TDC) is the key enzyme in serotonin biosynthesis, and SlTDC1 has been identified as a critical enzyme in tomatoes. Overexpression of *SlTDC1* significantly increases serotonin levels in transgenic plants (Kang *et al*., 2007; Tsunoda *et al*., 2021). Given the regulatory challenges associated with transgenic crops, developing non‐transgenic varieties enriched in serotonin is highly valuable. The success of gene‐edited tomatoes with elevated gamma‐aminobutyric acid (GABA) levels by Sanatech Seed in Japan (Waltz, 2022) highlights the potential of gene editing for specialty crop development.

Upstream open reading frames (uORFs) are critical translational regulators that fine‐tune gene expression by repressing main ORF translation under specific conditions (Zhang *et al*., 2021). In plants, uORFs have been shown to function in plant development, stress response, and nutrient biosynthesis (van der Horst *et al*., 2020; von Arnim *et al*., 2014), offering a precise tool for metabolic engineering. Here, we target three uORFs in the SlTDC1 promoter to derepress serotonin biosynthesis, leveraging CRISPR/Cas9 to develop transgene‐free nutritionally enhanced tomatoes.

Three uORFs were identified within the 541‐bp promoter region of *SlTDC1* (as detailed in Materials and Methods in Supporting Information) (Figure 1a). To investigate the translational repression activity of *SlTDC1* uORFs, a dual‐luciferase assay was performed in *N. benthamiana*. Wild‐type *SlTDC1 uORFs* and a mutated version carrying mutations in all three uORFs were cloned into the *35S‐GFP* vector, creating constructs *35S‐uORFs‐GFP* and *35S‐uorfs‐GFP* (Figure 1b and Figure S1). These constructs were infiltrated into *N. benthamiana* leaves, and GFP expression was measured using a fluorescence assay (Figure 1c). GFP protein levels were also determined by Western blot analysis (Figure 1d). The results revealed that WT uORFs of *SlTDC1* inhibited GFP translation compared to controls, while mutations in these uORFs (Δ7 bp in uORF1, Δ5 bp in uORF2, and Δ8 bp in uORF3) restored GFP expression (Figure 1c,d). This suggests that the uORFs act as translational repressors, and their disruption might upregulate *SlTDC1* expression.

**Figure 1:**
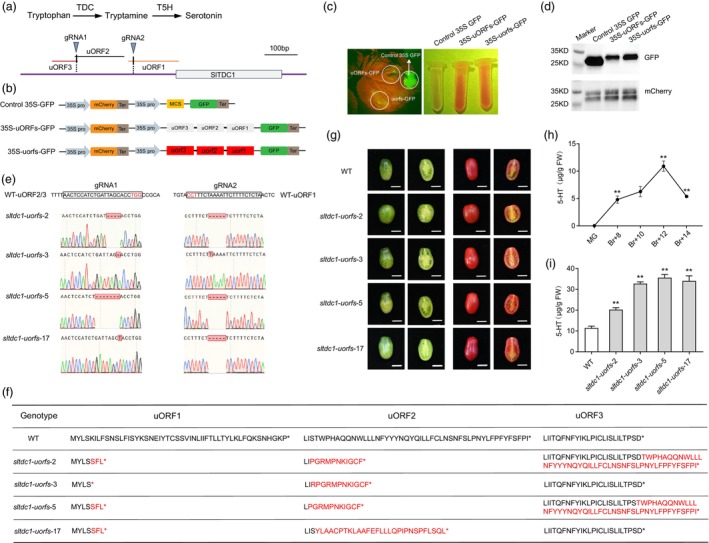
CRISPR/Cas9‐mediated editing of uORFs in *SlTDC1* enhances serotonin biosynthesis in tomato. (a) Serotonin biosynthesis pathway and schematic of the *SlTDC1* promoter region (541‐bp upstream of the ATG start codon) showing three uORFs (uORF1, uORF2, uORF3). (b) Construct designs for transient expression assays: 35S‐GFP (control), 35S‐uORFs‐GFP (wild‐type uORFs fused to GFP), and 35S‐uorfs‐GFP (mutated uORFs with deletions: Δ7 bp in uORF1, Δ5 bp in uORF2, Δ8 bp in uORF3). (c, d) GFP fluorescence (c) and Western blot (d) showing uORF‐mediated repression. (e) Genomic sequences of wild‐type (WT) and CRISPR‐edited *sltdc1‐uorf* lines (*sltdc1‐uorf‐2*, *‐3*, *‐5*, *‐17*). Dashed red boxes: deleted nucleotides. (f) Predicted amino acid sequences of uORFs in WT and mutant lines. (g) Phenotypes of WT and *sltdc1‐uorf* mutant fruits at mature green (MG) and Br + 12 stages. Scale bars: 1 cm. No significant morphological differences were observed. (h) Serotonin accumulation in WT fruits at five different stages (*n* = 4 fruits per stage). (i) Serotonin levels in Br + 12 fruits of WT and *sltdc1‐uorf* mutants. The statistical comparisons were made between WT and *sltdc1‐uorf* mutants. Data were shown as mean ± SD (*n* = 4); **P < 0.01, *P < 0.05 (Student's *t*‐test).

To test this hypothesis, we employed a dual‐gRNAs CRISPR/Cas9 system to target uORF1, uORF2, and uORF3 of SlTDC1 in tomato. Stable transformation via *Agrobacterium* resulted in four transgenic lines with homozygous mutations in the T2 generation (Figure 1e). Mutations included frame‐shifts in uORF1 and uORF2, and stop‐codon mutations in uORF3. Amino acid sequence alignments revealed that the mutations disrupted the predicted regulatory uORFs, likely abolishing uORF‐mediated repression (Figure 1f). Serotonin levels in WT tomatoes peaked at the Br + 12 stage of fruit development (Figure 1h). To investigate the role of *SlTDC1* uORFs in serotonin regulation, we examined the serotonin levels in four *sltdc1‐uorf* mutants (*sltdc1‐uorf‐2, ‐3, ‐5*, and ‐*17*) at the Br + 12 stage. In *sltdc1‐uorf* mutants, serotonin accumulation was significantly higher, with increases ranging from 1.8‐ to 3.1‐fold over the WT levels (Figure 1i). Notably, these mutations did not affect fruit growth or plant development (Figure 1g, Figure S2), indicating the potential for uORF editing to enhance nutritional value without compromising agronomic traits.

This study demonstrates that uORF editing can generate transgene‐free tomatoes with enhanced levels of the neurotransmitter serotonin. By disrupting *SlTDC1 uORFs*, we achieved serotonin levels comparable to transgenic overexpression lines (Kang *et al*., 2007), without compromising agronomic traits. The success of GABA‐enriched tomatoes and our uORF‐edited serotonin‐enriched lines highlights CRISPR's potential for regulatory‐compliant crop improvement for nutritional fortification. Future work could explore uORF editing in other metabolic pathways or combine multiple edits for synergistic benefits.

## Conflicts of interest

The authors declare no conflicts of interest.

## Author contributions

S. Z., L. Z., Y. L., and Q. N. performed the experiments. X. N., J.‐K. Z., and Z. L. designed the study and interpreted the data. S. Z., Q. N., J.‐K. Z., and Z. L. wrote the manuscript.

## Supporting information


**Figure S1** Sequences of 541‐bp upstream of SlTDC1, red letters represent deleted sequences. Deleted‐1 indicated deletion of 7 bp of uORF3 at the start, Deleted‐2 indicated deletion of 12 bp of uORF3 at the end and 5 bp of uORF2 at the start, Deleted‐3 indicated deletion of 8 bp of uORF1 at the start.
**Figure S2** The tomato plant of *sltdc1‐uorfs* mutants and the wild‐type in the flowering period.
**Table S1** Primers in this study.

## Data Availability

The data that support the findings of this study are available upon request from the corresponding author.
