# Supplementary material for: CRISPR/Cas9‐mediated editing of uORFs in the tryptophan decarboxylase gene SlTDC1 enhances serotonin biosynthesis in tomato
Source: Plant Biotechnol J. 2025 May 31;23(8):3392–4. doi: 10.1111/pbi.70106 (PMC12310824; doi:10.1111/pbi.70106)
Supplement: Supplementary file 1 — Figure S1 Sequences of 541‐bp upstream of SlTDC1, red letters represent deleted sequences. Deleted‐1 indicated deletion of 7 bp of uORF3 at the start, Deleted‐2 indicated deletion of 12 bp of uORF3 at the end and 5 bp of uORF2 at the start, Deleted‐3 indicated deletion of 8 bp of uORF1 at the start. Figure S2 The tomato plant of sltdc1‐uorfs mutants and the wild‐type in the flowering period. Table S1 Primers in this study. [file PBI-23-3392-s001.docx]

**Materials and Methods**

1. **Plant materials and growth conditions.**

For the generation of *sltdc1-uorfs* mutants, uORF of SlTDC1 (Solyc07g054860.1.1) identification was performed through Sequence analysis using using uORFscan (https://rnainformatics.org.cn/RiboUORF/uORFscan.php) with default parameters and employed the CRISPR/Cas9 editing system. The construction of the expression vector was performed as the author described. Briefly, 20-bp sequences upstream of protospacer-adjacent motif (PAM) were synthesized and constructed into sgRNA intermediate plasmids at BsaI site. All constructs were confirmed by sequencing and introduced into Agrobacterium tumefaciens EHA105 cells. The transformation into G17-60 was subsequently executed through Agrobacterium-mediated transformation. For the determination of mutations at the target sites, genomic DNA was extracted from leaves of the transgenic plants and PCR were performed to amplify the genomic region covering all of the uORFs. The mutation styles of transgenic plants were identified by comparing the sequenced results with that of WT plants with primers flanking the corresponding mutation sites. All primers used for vector construction are listed in (Table S1). All plants were grown in a growth room, with room temperature (25 ± 2◦C) and a 16 hours light/8 hours dark cycle.

1. **Transient expression assay**

The mutated uORFs of SlTDC1 and wild-type uORFs were inserted into the upstream GFP protein of the pCAMBIAsuper1300-GFP vector to perform a transient expression assay in *N. benthamiana* plants. Following this, the *N. benthamiana* plants were subjected to an incubation period of 24 hours under conditions of darkness at a temperature of 25 ℃. Subsequently, the plants were cultivated at room temperature (25 ℃) under a 16-hour light/8-hour dark cycle for 24 hours. The pCAMBIAsuper1300-GFP vector was utilized as a control. Finally, GFP fluorescence were scanned with a NightSHADE evo LB 985N (Berthold, Bad Wildbad, Germany). Three individual plants were infiltrated as biological replicates. Primer sequences are detailed in Table S1.

1. **Protein extraction and immunoblotting**

For protein extraction from the leaves and petals of each cultivar, total protein was isolated with extraction buffer containing 50 mmol L^-1^ Tris-HCl (pH = 7.5), 0.1% Triton X-100, 150 mmol L^- 1^ NaCl, 1 mmol L^-1^EDTA, 10% glycerol and 1 protease inhibitor cocktail (Roche, Mannheim, Germany). GFP fluorescence of each protein extract in 1.5 ml tubes were scanned with a NightSHADE evo LB 985N (Berthold, Bad Wildbad, Germany). Protein concentration of each extract was determined using a BCA protein assay kit (Sangon, Shanghai, China). For the immunoblotting assays, total proteins were separated by SDS–PAGE on 10% polyacrylamide gels and transferred to 0.22 μm Immobilon® -FL PVDF Membrane (Merck KGaA, Darmstadt, Germany). Antibodies of GFP (ABclonal, Wuhan, China) or Actin ((ABclonal, Wuhan, China) were used by dilution for 1000-fold, which was then marked by goat anti-rabbit IgG (ABclonal, Wuhan, China) conjugated with horseradish peroxidase by dilution for 10,000-fold. and photograph was taken by using a Canon EOS Kiss Digital X7i camera under UV LED lights as described above.

1. **Determination of serotonin content in fresh**

The extraction of serotonin from tomato fruit at Break+12 day was performed as follows: Approximately 10 mg of finely ground fruit material was homogenized with 1 mL of ultrapure water and ultrasonicated at room temperature for 30 min. The mixture was centrifuged, and the supernatant was filtered through a 0.22 μm organic phase membrane. Quantification was conducted via high-performance liquid chromatography-tandem mass spectrometry (HPLC-MS/MS) (Thermo Fisher Scientific, MA, USA) using a BEH C18 column with a mobile phase of 0.1% formic acid in water (A) and 0.1% formic acid in acetonitrile (B) at a flow rate of 0.3 mL/min and column temperature of 40℃. Results were calculated based on peak areas and expressed as μg serotonin per kg sample (μg/kg), normalized to the initial sample mass.


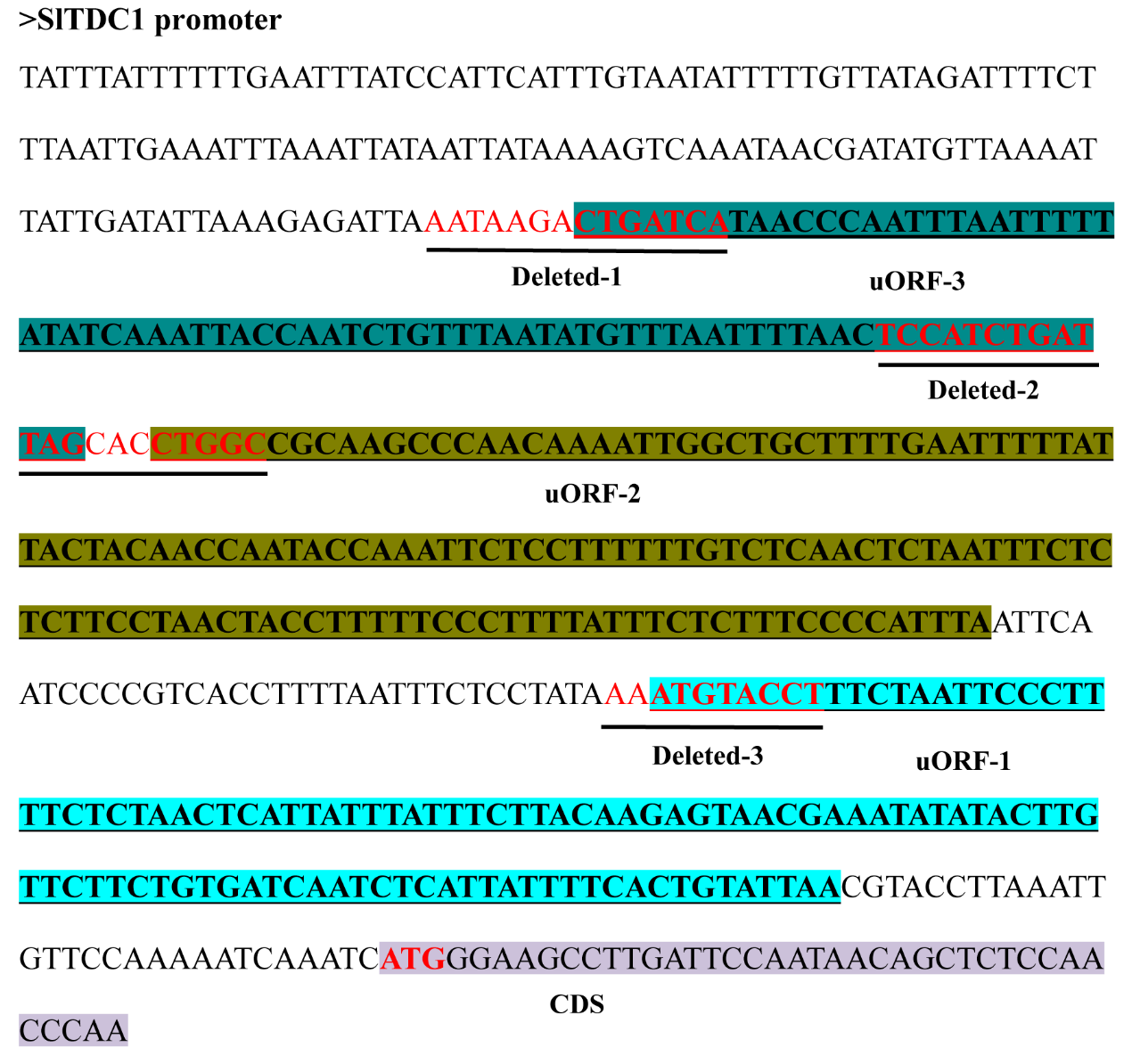


**Supplementary Fig. 1** Sequences of 541 bp upstream of SlTDC1, red letters represent deleted sequences. Deleted-1 indicated deleted 7bp of uORF3 at the start, Deleted-2 indicated deleted 12bp of uORF3 at the end and 5bp of uORF2 at the start, Deleted-3 indicated deleted 8bp of uORF1 at the start.


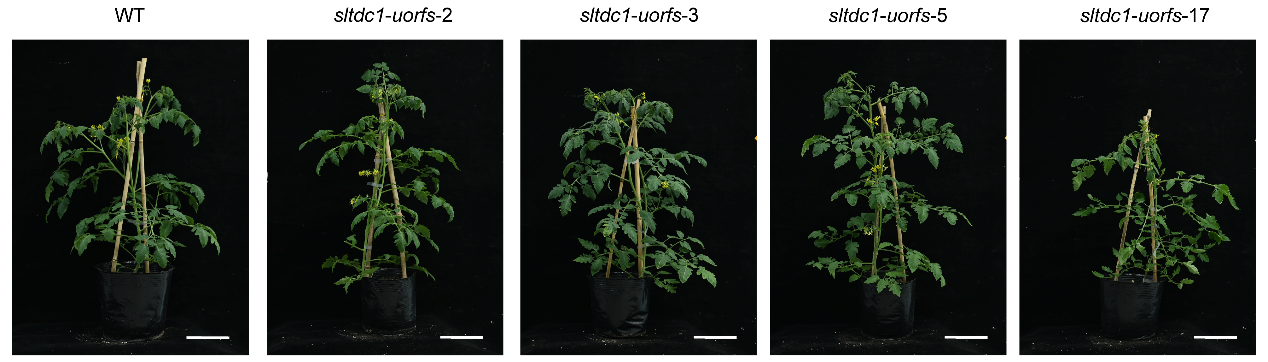


**Supplementary Fig. 2** The tomato plant of *sltdc1-uorfs* mutants and the wild-type in the flowering period.

**Supplementary Table 1.** Primers in this study

| Name | Primers | Application |
| --- | --- | --- |
| SlTDC1-uORF-sg1-F | TGATTGAACTCCATCTGATTAGCACC | CRISPR  /Cas9 |
| SlTDC1-uORF-sg1-R | AAACGGTGCTAATCAGATGGAGTTCA |  |
| SlTDC1-uORF-sg2-F | TGATTGTTCTAAAATTCTTTTCTCTA |  |
| SlTDC1-uORF-sg2-R | AAACTAGAGAAAAGAATTTTAGAACA |  |
| SlTDC1-p-F | TTTTTTTTTAAATCCAGTGCCA | identification |
| SlTDC1-p-R | TCCGGGTCAAGCGGGTTGAATT |  |
| SlTDC1-uORFs -F | ggacagggtacccggggatccTATTTATTTTTTGAATTTA | pCAMBIAsuper  1300-GFP |
| SlTDC1-uORFs -F | gacggcgcgcctcgaactagtGATTTGATTTTTGGAAC |  |
